# Assets, stressors, and symptoms of persistent depression over the first year of the COVID-19 pandemic

**DOI:** 10.1126/sciadv.abm9737

**Published:** 2022-03-02

**Authors:** Catherine K. Ettman, Gregory H. Cohen, Salma M. Abdalla, Ludovic Trinquart, Brian C. Castrucci, Rachel H. Bork, Melissa A. Clark, Ira B. Wilson, Patrick M. Vivier, Sandro Galea

**Affiliations:** 1 Boston University School of Public Health, Boston, MA, USA.; 2 Brown University School of Public Health, Providence, RI, USA.; 3 Institute for Clinical Research and Health Policy Studies, Tufts Medical Center, Boston, Massachusetts, USA.; 4 Tufts Clinical and Translational Science Institute, Tufts University, Boston, Massachusetts, USA.; 5 de Beaumont Foundation, Bethesda, MD, USA.; 6 Hassenfeld Child Health Innovation Institute, Providence, RI, USA.

## Abstract

The coronavirus disease 2019 (COVID-19) pandemic has been accompanied by an increase in depression in U.S. adults. Previous literature suggests that having assets may protect against depression. Using a nationally representative longitudinal panel survey of U.S. adults studied in March and April 2020 and in March and April 2021, we found that (i) 20.3% of U.S. adults reported symptoms of persistent depression in Spring 2020 and Spring 2021, (ii) having more assets was associated with lower symptoms of persistent depression, with financial assets—household income and savings—most strongly associated, and (iii) while having assets appeared to protect persons—in particular those without stressors—from symptoms of persistent depression over the COVID-19 pandemic, having assets did not appear to reduce the effects of job loss, financial difficulties, or relationship stress on symptoms of persistent depression. Efforts to reduce population depression should consider the role played by assets in shaping risk of symptoms of persistent depression.

## Teaser

This work highlights how assets shaped mental health and how inequities were exacerbated during the COVID-19 pandemic.

## INTRODUCTION

The first year of the coronavirus disease 2019 (COVID-19) pandemic presented unprecedented challenges for population mental health. The threat and fear of a new devastating infectious disease (*1*), millions of deaths globally, and unprecedented reductions in social interactions were each stressors that could be expected to influence mental health. In addition, the efforts to mitigate the pandemic were accompanied by an economic downturn that contributed to poor mental health (*2*–*5*). An increase in poor mental health at the start of the COVID-19 pandemic relative to before it has been documented across multiple studies. Daly *et al.* (*6*) reported an increase in symptoms of depression from 8.7% in 2017 to 2018 to 14.4% in April 2020 using the Patient Health Questionnaire-2 (PHQ-2) screener for depressive symptoms. Czeisler *et al.* (*7*) documented a population prevalence of 24.3% for symptoms of depressive disorder in June 2020 also using the PHQ-2. Using the nine-question PHQ-9, Ettman *et al.* (*8*) reported an increase in elevated symptoms of probable depression from 8.5% in 2017 to 2018 to 27.8% in March and April 2020, suggesting a potential threefold increase in symptoms of probable depression at the start of the COVID-19 pandemic. McGinty *et al.* (*9*) measured symptoms of serious psychological distress using the Kessler 6 Psychological Distress Scale and documented in an increase from 3.9% in 2018 to 13.6% in April 2020, suggesting a 3.5-fold increase in symptoms of psychological distress. While the increase in depression at the onset of the pandemic may not have been unexpected given what we knew about the risks for depression before the pandemic, depression remained high through the end of 2020 as the COVID-19 pandemic continued (*10*). Vahratian *et al.* (*10*) reported a continued increase in symptoms of depressive disorder from 24.5% in August 2020 to 30.2% in December 2020. Ettman *et al.* (*11*) reported that 32.8% of U.S. adults reported elevated symptoms of probable depression in March and April 2021.

The continued high prevalence of depression is unusual. In the aftermath of other mass traumatic events, population mental health improved in the months that followed the large-scale trauma. For example, after an initial increase, population depression decreased substantially in the first six months after Hurricane Ike (*12*), the 1999 Mexico floods (*13*), and the September 11th attacks (*13*, *14*). The chronic and continued exposure to the COVID-19 pandemic throughout 2020 may have resulted in the observed persistence of high levels of depression over time at the population level during the pandemic. Comparison with population mental health following the last pandemic of similar scale, namely, the 1918 Flu Pandemic, is challenging, given advances in the field on mental health screening instruments and classification of conditions. Even so, publication from the time suggested that while around one-fourth of cases at a Boston-based hospital showed depression at any time, it was not chronic or persistent when present (*15*). Persistent depression among individuals (that is, unrelenting depression, or depression expressed by the same person across multiple times) is particularly concerning given its potential for ongoing health and economic consequences in populations (*16*). It was estimated that depression cost the United States over $210 billion in 2010 including absenteeism (missed work), presenteeism (underproduction while at work), and costs for treatment, among others (*17*). The economic toll of depression could be considerably larger with an increased prevalence of the population reporting symptoms of depression.

The role of assets may be essential to understanding the persistently high burden of depression in the population during the COVID-19 pandemic. Assets can protect against poor mental health, as noted before the COVID-19 pandemic (*18*–*20*). In particular, financial assets, physical assets, and social assets may all protect against depression (*19*, *21*). For example, having family savings was associated with 150% greater odds of symptoms of depression relative to not having family savings in 2015 to 2016 (*18*). Beyond just having family savings, owning a home was also associated with lower odds of symptoms of depression: Homeowners without savings had 2.15 times the odds of symptoms of depression relative to homeowners with $5000 in family savings, and home renters without family savings had 3.65 times the odds of symptoms of depression relative to home renters with home savings (*20*). The association of assets with probable depression is so strong that it may, in fact, account for much of the difference in population-level depression between racial-ethnic groups (*19*). Having more assets was associated with a lower prevalence of probable depression at the start of COVID-19, including in the face of stressors (*22*). It is possible that having access to assets may also protect persons against persistent or chronic depression over time. We do not know the prevalence of symptoms of persistent depression, nor do we know the factors associated with greater risk for symptoms of persistent depression following exposure to stressors after the presence of the COVID-19 pandemic for 1 year.

Therefore, in this work, we aimed to understand the following: (i) the population prevalence of symptoms of persistent depression at two time points, 1 year apart, during the COVID-19 pandemic; (ii) the relative influence of particular types of assets on symptoms of persistent depression over that time; and (iii) whether having assets reduced the effect of stressors on symptoms of persistent depression over the course of the COVID-19 pandemic. This paper addresses gaps in the literature by using a nationally representative, longitudinal panel study to measure symptoms of persistent depression 1 year into the COVID-19 pandemic, measuring symptoms of persistent depression in March and April 2020 and in March and April 2021 in U.S. adults. We use detailed assets and stressor exposures measured at the start of the COVID-19 pandemic to predict symptoms of persistent depression 1 year later.

## RESULTS

Table 1 shows the prevalence of symptoms of persistent depression by gender, age, and race/ethnicity. Twenty percent of U.S. adults reported symptoms of persistent depression, reporting elevated symptoms of probable depression in both March and April 2020 and March and April 2021. Among women, 24.8% reported symptoms of persistent depression and among men, 15.4% reported symptoms of persistent depression (*P* < 0.01). Persons ages 18 to 39 years reported the highest prevalence of symptoms of persistent depression (26.8%) relative to persons ages 40 to 59 years (20.7%) and persons ages 60 years and older (10.2%) (*P* < 0.01). We found no evidence of differences in the prevalence of symptoms of persistent depression across race/ethnicity.

**Table 1.** Symptoms of persistent depression in March and April 2020 (T1) and March and April 2021 (T2) by gender, age, and race/ethnicity. Note: T1 demographic characteristics reported. Other race includes multiple races and non-Hispanic Asian race. Column percentages provided for total; row percentages provided for persistent depression. Symptoms of persistent depression defined as presence of PHQ-9 score of 10 or greater at T1 and T2. *n* unweighted, % weighted using T2 survey weights. *P* value reflects the two-sided χ^2^ test between persistent depression and all other categories (people with no depression, depression only at T1, or depression only at T2). *P* values <0.05 suggest significance in differences between persistent depression and all other categories by demographic characteristics.

	**Total**		**Symptoms of persistent** **depression**		**All other categories**		
	***n***	**%**	***n***	**%**	***n***	**%**	***P* value**
**Total**	1139		208	20.3	931	79.7	
**Gender**							0.008
Female	563	51.8	130	24.8	433	75.2	
Male	576	48.2	78	15.4	498	84.6	
**Age**							<0.001
18–39 years	458	40.3	102	26.8	356	73.2	
40–59 years	380	32.0	76	20.7	304	79.3	
≥60 years	301	27.7	30	10.2	271	89.8	
**Race/ethnicity**							0.602
Black, non- Hispanic	95	11.9	13	16.2	82	83.8	
Hispanic, any race or races	186	16.4	39	23.9	147	76.1	
White, non- Hispanic	773	63.1	139	20.7	634	79.3	
Other race, non-Hispanic	85	8.6	17	15.8	68	84.2	

**Fig. 1.**
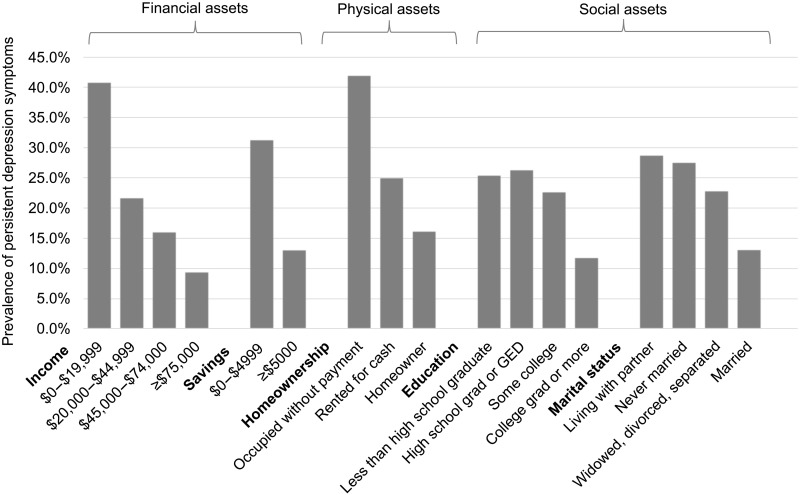
Prevalence of symptoms of persistent depression in March and April 2021 (T2) by financial assets, physical assets, and social assets in March and April 2020 (T1). Note: T1 assets reported. Symptoms of persistent depression defined as presence of PHQ-9 score of 10 or greater at T1 and T2. GED, graduate equivalency degree/general educational diploma. Percentages weighted using T2 survey weights.

Figure 1 shows a visual representation of the prevalence of symptoms of persistent depression by three types of assets, which are described in greater detail in Materials and Methods: financial assets, physical assets, and social assets. Financial assets include household income and household savings; physical assets include homeownership; and social assets include educational attainment and marital status, as published previously (*19*). The graph shows that as each asset type increased, the prevalence of persistent depression decreased. While persons with more social assets reported lower prevalence of depression than persons with fewer social assets, the spread between the groups was greatest between high- and low-asset holders for financial assets and physical assets. Persistent depression was highest among persons with low household income, with 40.9% of persons with $0 to $19,999 and 9.5% of persons with $75,000 or more in annual household income reporting persistent depression (*P* < 0.01). Thirty-one percent of persons with less than $5000 in household savings relative to 13.2% of persons with $5000 or more in household savings reported persistent depression (*P* < 0.01). Twenty-five percent of home renters and 16.3% of homeowners reported persistent depression (*P* < 0.01). More than 25% percent of persons without a high school degree reported persistent depression, while 11.9% of persons with a college degree or higher reported persistent depression. Persons who were not married reported a higher prevalence of persistent depression than persons who were married; 28% of persons who had never married and 28% of those who were living with a partner versus 13.2% of persons who were married reported persistent depression.

Table 2 shows the odds of symptoms of persistent depression by financial assets (income and savings), physical assets (homeownership), and social assets (education and marital status). Persons with a household income of $0 to $19,999 relative to $75,000 or more had 6.8 times the odds of symptoms of persistent depression, adjusting for demographic characteristics (model 2). Persons with less than relative to more than $5000 in household savings had 2.7 times the odds of symptoms of persistent depression when adjusting for demographics (model 3). Persons with a high school degree or graduate equivalency degree/general educational diploma (GED) had 2.9 times the odds of symptoms of persistent depression as persons with a college degree or more (model 5). Persons who were never married had 2.1 times the odds and persons who were widowed, divorced, or separated had 2.0 times the odds of symptoms of persistent depression as persons who were married, when controlling for demographics (model 6). Model 7 shows the adjusted odds of symptoms of persistent depression, adjusting for all assets (which are correlated with each other; see table S1) and demographic characteristics. When adjusting for financial, physical, and social assets at the same time, having a household income of $0 to $19,999 relative to $75,000 or more was associated with 3.5 times the odds of symptoms of persistent depression and having household savings of less than $5000 was associated with 1.7 times the odds of symptoms of persistent depression.

**Table 2.** Odds ratios of symptoms of persistent depression in March and April 2021 (T2) by assets in March and April 2020 (T1). Note: Odds radio (OR), adjusted odds ratios (aOR), and 95% confidence interval (CI) presented. Model 1: unadjusted. Models 2 to 6: adjusted for household income, household savings, homeownership, education, or marital status, respectively, and gender, age, race/ethnicity, and household size. Model 7: multivariable model adjusted for gender, age, race/ethnicity, household size, and all assets (household income, household savings, homeownership, education, and marital status). Symptoms of persistent depression defined as presence of the PHQ-9 score of 10 or greater at T1 and T2. Data weighted using T2 survey weights. Ref, reference.

	**Model 1**	**Model 2**	**Model 3**	**Model 4**	**Model 5**	**Model 6**	**Model 7**
	OR (95% CI)	aOR (95% CI)	aOR (95% CI)	aOR (95% CI)	aOR (95% CI)	aOR (95% CI)	aOR (95% CI)
**Household income**							
$0–$19,999	6.6 (3.5–12.2)	6.8 (3.7–12.6)	–	–	–	–	3.5 (1.6–7.6)
$20,000–$44,999	2.6 (1.5–4.6)	2.7 (1.5–4.9)	–	–	–	–	1.7 (0.9–3.3)
$45,000–$74,999	1.8 (1.0–3.4)	1.8 (1.0–3.2)	–	–	–	–	1.3 (0.7–2.4)
≥$75,000	Ref	Ref	–	–	–	–	
**Household savings**							
$0–$4999	3.0 (1.9–4.7)	–	2.7 (1.7–4.2)	–	–	–	1.7 (1.0–2.8)
≥$5000		–	Ref	–	–	–	
**Homeownership**							
Occupied without payment	3.7 (1.1–2.6)	–	–	3.1 (1.3–7.5)	–	–	0.9 (0.5–1.5)
Rented for cash	1.7 (1.4–9.9)	–	–	1.4 (0.9–2.3)	–	–	1.6 (0.6–4.2)
Homeowner		–	–	Ref	–	–	
**Education**							
Less than high school graduate	2.5 (1.0–6.6)	–	–	–	2.6 (1.0–6.6)	–	1.1 (0.4–2.8)
High school graduate or GED	2.7 (1.6–4.5)	–	–	–	2.9 (1.7–5.1)	–	1.6 (0.8–3.0)
Some college	2.2 (1.4–3.4)	–	–	–	2.2 (1.4–3.5)	–	1.6 (0.9–2.6)
College graduate or more		–	–	–	Ref	–	
**Marital status**							
Living with partner	2.7 (1.3–5.5)	–	–	–	–	2.0 (1.0–4.0)	1.4 (0.7–2.9)
Never married	2.5 (1.5–4.2)	–	–	–	–	2.1 (1.3–3.5)	1.5 (0.8–2.7)
Widowed, divorced, separated	2.0 (1.2–3.2)	–	–	–	–	2.0 (1.2–3.5)	1.5 (0.8–2.7)
Married		–	–	–	–		

**Fig. 2.**
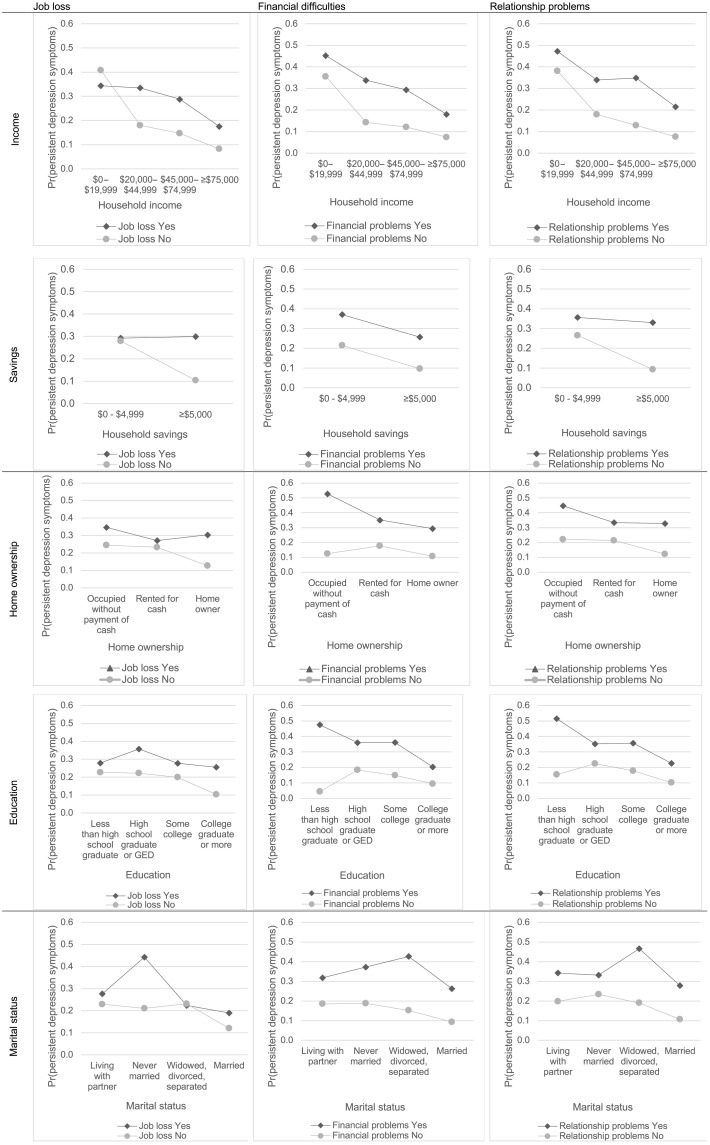
Predicted probability of symptoms of persistent depression in March and April 2021 (T2) by stressors and assets in March and April 2020 (T1). Note: T1 stressors and assets reported. Symptoms of persistent depression defined as presence of the PHQ-9 score of 10 or greater at T1 and T2. Models adjusted for gender, age, race/ethnicity, and household size. Unweighted.

Figure 2 shows the predicted probability of symptoms of persistent depression adjusted for demographic characteristics and for the interaction of job loss, financial difficulties, and relationship problems with each asset type. Assets did not appear to reduce the effect of job loss, financial difficulties, or relationship problems on symptoms of persistent depression. Assets were, however, associated with lower symptoms of persistent depression among persons who did not report stressors. In particular, persons who had more than $5000 in savings and did not report job loss had substantially lower symptoms of persistent depression than persons who had less than $5000 in savings and did not report job loss; similarly, persons with $5000 or more in savings and did not report relationship problems had substantially lower symptoms of persistent depression than their counterparts with less than $5000 in savings.

Table S1 shows the correlation of assets with each another, and table S2 shows the prevalence of symptoms of persistent depression by financial assets, physical assets, and social assets. Table S3 shows the adjusted predicted probabilities and 95% confidence intervals (CIs) for all interaction pairs. Table S4 shows the results of likelihood ratio tests for the interaction between each stressor (job loss, financial problems, and relationship problems) and each asset type. At the 0.05 level of significance, there was evidence of interaction between savings and job loss and between savings and relationship problems on symptoms of persistent depression. Figure S1 shows the unadjusted, unweighted prevalence of symptoms of persistent depression across each stressor and asset type combination.

## DISCUSSION

In a nationally representative longitudinal panel study of U.S. adults 1 year into the COVID-19 pandemic, we describe three main findings: (i) 20.3% of surveyed U.S. adults reported symptoms of depression in both March and April 2020 and March and April 2021; (ii) financial assets, physical assets, and social assets were each associated with a lower likelihood of symptoms of persistent depression 1 year into the COVID-19 pandemic, with the strongest associations among financial assets; and (iii) persons with fewer assets and more stressors in March and April 2020 were more likely to report symptoms of persistent depression 1 year later, in March and April 2021, controlling for gender, race/ethnicity, age, and household size. Having assets was particularly important for reducing symptoms of persistent depression 1 year into the COVID-19 pandemic in the absence of stressors. People who experienced stressors had greater symptoms of persistent depression than persons who did not. Persons with lowest risk of symptoms of persistent depression in Spring 2021 were those with high assets in Spring 2020 and no exposure to job loss, financial difficulties, or relationship problems.

These findings are consistent with some but not all other studies that have reported a consistently high prevalence of symptoms of depression during the COVID-19 pandemic in population-level longitudinal cohorts. Most of the studies published to date on longitudinal cohorts have been conducted outside of the United States, in Australia, Austria, and the United Kingdom, among others. Czeisler *et al.* (*23*) found an unchanged prevalence of probable depression in the Australian population in April 2020 and September 2020. However, Australia maintained its lockdown during the two survey periods, unlike the United States, whose COVID-19 restriction policies were largely being lifted around the timing of the COVID-19 and Life stressors Impact on Mental Health and Well-being (CLIMB) Time 2 (T2) survey. In Austria, which did lift its lockdown policies before the CLIMB T2 collection, Pieh *et al.* (*24*) found no significant change in probable depression between April 2020 and September 2020; they found that 18.3 and 19.7% of the sample reported PHQ-9 scores of 10 or greater at T1 and T2, respectively. Several studies conducted on longitudinal cohorts in the United Kingdom reported that depressive symptoms improved after the initial start of the COVID-19 pandemic. Fancourt *et al.* (*25*) and Pierce *et al.* (*26*) reported that depressive symptoms improved from the start of the COVID-19 pandemic and August 2020 and October 2020, respectively. However, these studies were conducted during summer months, which may have been confounded by seasonal effects resulting in improved affect. In addition, a requirement that participants provide at least three repeated measures (i.e., participants had to respond to at least three of the weekly surveys between 23 March 2020 and 9 August 2020 to be included in the sample) in the study by Fancourt *et al.* may be susceptible to survivorship bias (*27*), which could lead to an underreporting of adverse mental health symptoms at the population level. Given that the CLIMB survey had a response rate of 81.1% at T2, our data may be less susceptible to survivorship bias.

Within the United States, there are few studies that have longitudinally followed populations during the COVID-19 pandemic. While several studies compared adult depressive symptoms relative to the start of the COVID-19 pandemic (*6*, *28*, *29*), none to our knowledge has reported on persistent depression (presence of repeatedly reporting probable depression in the same persons) reported out as far as April 2021. Of the longitudinal studies conducted at the start of the COVID-19 pandemic, findings suggested no change or a slight decline in depressive symptoms during the first half of 2020 (*30*–*33*). For example, Shuster *et al.* (*32*) found that anxiety and depressive symptoms declined after the initial weeks of COVID-19, measured between April 2020 and June 2020. Their study differs from ours in that the population was not representative of U.S. adults, captured a 10-week span (relative to our 12-month comparison), and may have seen a decline in depressive symptoms due to loss to follow-up of persons with depression and seasonal effects, with affect improving during summer months. Our findings were consistent with theirs in that they reported that female gender, younger age, and lower household income were associated with increased depression across time. They also found that worsening economic situation due to COVID-19 was associated with increased depression over time (*32*). The most recent longitudinal study of which we are aware measured depressive symptoms over the previous 7 days using the PHQ-2 with the last reporting period being from 20 January to 1 February 2021 (*10*). While the authors used an abbreviated form of the PHQ-9 and did not capture detailed asset or stressor information, they reported an increase in the percentage of adults with recent symptoms of an anxiety or depressive disorder (*10*), with 30.2% of U.S. adults reporting symptoms of a depressive disorder as of January and February 2021 (relative to our finding that 20.3% of U.S. adults reported symptoms of depressive disorder at both March and April 2020 and March and April 2021). According to the Centers for Disease Control National Center for Health Statistics Household Pulse Survey, which used the shorter-form PHQ-2, 24.7% of U.S. adults reported symptoms of depressive disorder between 17 March and 29 March 2021, which closely aligned with our survey collection at T2 (*34*).

Our findings were consistent with other studies that have addressed the stressors of job loss, financial strain, and relationship conflict during COVID-19. In a cross-sectional study conducted in April 2020, McDowell *et al.* (*35*) reported an increase in symptoms of depression among persons who reported job loss. Hertz-Palmor *et al.* (*36*) assessed relations between financial strain and depressive symptoms in March and April 2020 and 1 month later in samples in the United States and Israel. They found that income loss and financial strain were associated with exacerbated depressive symptoms in their 1-month follow-up sample. Lee *et al.* (*37*) studied relationship conflict during COVID-19 from March and April 2020 and found that in the weeks studied, relationship conflict increased. Although their findings did not show a significant association between relationship conflict and depressive symptoms, this may have been due to limitations in sample size (*N* = 291) (*37*). Nonetheless, these studies show early evidence that exposure to stressors during the COVID-19 pandemic was associated with depressive symptoms.

Our findings that having more assets was associated with lower depression are novel in the context of COVID-19, even if consistent with studies conducted after other traumatic and stressful events. For example, Gallo *et al.* (*38*) found that persistent depression lowered over time following involuntary job loss but remained highest among low-wealth persons. Tracy *et al.* (*39*) found that low–socioeconomic status persons were more likely to report depressive symptoms following Hurricane Ike. Thus, although the conditions of COVID-19 were unique, these findings provide support for the notion that economic conditions can buffer the effects of stressors on depression. The COVID-19 pandemic in particular was unique in its wide-ranging scope, its ongoing nature, and the economic inequities that it produced (*40*). As a result, persistent depression may be higher 1 year into the COVID-19 pandemic than that documented after other large-scale events.

This study has three main limitations. First, the PHQ-9, which was used to assess symptoms of probable depression at each time point, is a depression screener, which does not replace the gold standard of clinical diagnosis. However, given a sensitivity and specificity of 88% relative to clinical diagnosis, the PHQ-9 is the best available measure that allows large-scale assessments to provide estimates of symptoms of depression consistent with probable depression at the national level across time (*41*). Using the PHQ-9 allowed us to estimate the burden of depression at the population level at the beginning of the pandemic, setting a baseline for symptoms of persistent depression, which was defined as presence of probable depression at T1 and T2. The study was not designed to measure continuous probable depression throughout the 12-month follow-up period but rather to measure presence of probable depression at both T1 and T2. Second, similar to all longitudinal studies following the same persons over time, we experienced some loss to follow-up at T2. However, with 81.1% of respondents replying at T2, we had a relatively high response rate, particularly given potential for survey response fatigue during the COVID-19 pandemic. It is possible that nonresponders at T2 had a higher prevalence of depression at T2 than responders (*27*), suggesting that the documented symptoms of persistent depression presented may represent an underestimate of the prevalence of probable depression at T2 and therefore symptoms of persistent depression. Survey weights accounting for nonresponse at T2 were used, allaying these concerns. Third, our sample size may have limited our ability to detect significant associations in the interactive effects of assets in protecting against persistent depression across stressor groups. However, that we were able to detect significant associations in the interactions of savings with relationship problems and job loss speaks to the magnitude of these stressors on poor mental health.

We found a high prevalence of persistent depression across a nationally representative group of U.S. adults measured longitudinally after 1 year of follow-up during the COVID-19 pandemic through April 2021. Our results highlight the importance of assets as a potential protective mechanism against ongoing probable depression. Exposure to stressors and having fewer assets were both associated with greater persistent depression 12 months into the COVID-19 pandemic. While assets did not appear to reduce the effect of stressors on persistent depression, having more assets and not experiencing stressors was associated with significantly less persistent depression than having fewer assets. These findings highlight the deleterious effect of stressors on mental health and the potential protective effect of assets against the COVID-19 pandemic in the absence of reported job loss, financial problems, and relationship stressors.

Given the high prevalence of depression in U.S. adults 1 year into the COVID-19 pandemic, with one in five surveyed U.S. adults screening positive for symptoms of probable depression both in March and April 2020 and in March and April 2021, finding multiple ways to address and mitigate the burden of poor mental health will be critical. These findings suggest that interventions to shore up economic contexts that people live in, in particular bolstering financial assets and reducing stressors, may serve to lessen persistent depression over time. Efforts to improve the economic status of low-asset populations may lead to improved mental health.

## MATERIALS AND METHODS

### Experimental design

This study used a nationally representative sample of U.S. residents ages 18 years and older followed longitudinally over 1 year of the COVID-19 pandemic. Participants were surveyed in March and April 2020 (T1) and in March and April 2021 (T2) as part of the CLIMB study (*11*). Participants were drawn from the AmeriSpeak panel, which is a nationally representative standing panel whose sampling frame covered 97% of U.S. households and used a two-stage probability-based sample design to recruit members. The AmeriSpeak panel has a household response rate of 34.2% (*42*). Participants provided consent at induction into the AmeriSpeak panel and at the beginning of each CLIMB survey. Participants were contacted over email and over the telephone if they did not respond to email outreach. The CLIMB survey completion rate for participants at T1 was 64.3% (*8*), and among those participants, the response rate at T2 was 81.1% (*11*). Details on sampling, demographics, and characteristics can be found in other published work (*8*, *11*, *22*, *43*). The final analytic sample in this paper included 1139 participants who responded to all depression questions at T1 and T2. Forty-four persons were removed from the analysis because they did not respond to all depression questions at T1 and/or T2. The participants who were not included in the analysis because of missing depression values did not differ significantly across gender, race/ethnicity, marital status, age, education, or household income status from participants who were included in the analysis. Survey weights accounted for nonresponse at T1 and at T2 and aligned the sample with the U.S. adult population according to the 2010 U.S. Census (*1*) using the following variables: age, gender, Census Division, race/ethnicity, education, housing type, and household phone status. The institutional review boards at NORC at the University of Chicago and Boston University approved this study.

### Key covariates

#### *Symptoms of persistent depression*

Participants completed the PHQ-9, a validated screening tool for depression at both T1 and T2. The PHQ-9 is a nine-item screening tool measuring probable depression based on the DSM-IV: Diagnostic and Statistical Manual of Mental Disorders, fourth edition. Participants responded to nine questions about their affect over the last 2 weeks; responses were tallied for corresponding scores ranging from 0 to 27. Using a PHQ-9 score of 10 or greater has a sensitivity of 88% and a specificity of 88% when tested against clinical diagnosis of depression (*41*). Having symptoms of persistent depression was defined as the presence of a PHQ-9 score of 10 or greater at both T1 and T2. We compared persons with persistent depression to all others in the sample, given the increased burden of poor mental health for persons reporting probable depression chronically across multiple time points (*16*). Because the PHQ-9 is a screening tool, it cannot replace official diagnosis of depression by a clinician (*44*). Throughout this paper, symptoms of persistent depression refer to the presence of elevated symptoms of probable depression in both March and April 2020 and March and April 2021 as indicated by a PHQ-9 score of 10 or greater at both times. This definition differs from persistent depressive disorder, reflecting the DSM-5 concepts of dysthymia and chronic major depression, which would be diagnosed by a clinician (*45*). Here, symptoms of persistent depression reflect the presence of elevated symptoms of probable depression at two points one year apart during the COVID-19 pandemic.

### Assets

To consider the role different types of assets, assets were grouped into three categories: financial assets, physical assets, and social assets, as previously published (*19*). Financial assets included household income and household savings. Household income was defined as a categorical variable: $0 to $19,999, $20,000 to $44,999, $45,000 to $74,999, and ≥$75,000 (*11*), with categories divided roughly at the interquartile range. To determine household savings, participants were asked to list total money in all types of accounts, including “cash, savings, or checking accounts, stocks, bonds, mutual funds, retirement funds (such as pensions, IRAs, 401Ks, etc.), and certificates of deposit” as consistent with national surveys (*18*). A binary variable was then created: $0 to $4999 or $5000 or more, as used previously (*8*, *11*, *22*). Physical assets referred to homeownership, which was defined as a categorical variable: homeowner, rented for cash, and occupied without payment of cash rent (*11*). Social assets included educational attainment and marital status. Educational attainment was defined as a categorical variable: less than high school graduate, high school graduate or GED, some college, including vocational/tech school, and college graduate or more (*11*). Marital status was defined as a categorical variable: married; widowed, divorced, or separated; never married; and living with partner (*11*). Assets at T1 were reported.

### Demographic characteristics

Key demographic characteristics include gender (female/male), age category (18 to 39 years, 40 to 59 years, and 60 years or older), race/ethnicity (Hispanic, non-Hispanic black, non-Hispanic white, and other race, including non-Hispanic Asian and multiple races), and household size (continuous variable, capped at 7).

### Stressors

To examine the role of assets in protecting against persistent depression, including in the face of stressors, we selected three stressors as examples of COVID-19–induced stressors experienced during the pandemic. We also aimed to understand whether assets modified the relation between stressors and persistent depression. We used three stressors that were highly associated with probable depression and, therefore, were candidates for potential effect measure modification. Job loss, financial difficulties, and relationship problems were each defined as a binary variable in response to the following question: “Have any of the following affected your life as a result of the coronavirus or COVID-19 outbreak?” Responses included the following: “losing a job,” “having financial problems,” and “family or relationship problems (for example, with your spouse or kids).” Stressors at T1 were reported.

### Statistical analysis

First, we calculated the prevalence of symptoms of persistent depression by gender, age, and race/ethnicity. Prevalence measures of symptoms of persistent depression were weighted unless otherwise noted. We used complex probability weights to account for nonresponse at T1 and T2 and to align with the U.S. adult population; the survey weights allowed for estimates to represent the U.S. national adult population (*11*). We conducted two-tailed χ^2^ analyses to measure the difference in distribution of symptoms of persistent depression across groups. Significance was set at *P* < 0.05. Demographic and asset variables at T1 were used to predict symptoms of persistent depression across time. Second, we calculated the weighted prevalence and odds along with their 95% CIs of symptoms of persistent depression across financial assets, physical assets, and social assets. Model 1 reported the unadjusted odds of symptoms of persistent depression by each asset; thus, model 1 showed the bivariable relation between symptoms of persistent depression and each asset, unadjusted for any other variable. Models 2 through 6 reported the odds of symptoms of persistent depression by each asset type, adjusting for demographic characteristics of age, gender, race/ethnicity, and household size (to account for sharing of assets within a household). Thus, models 2 to 6 show the adjusted odds of symptoms of persistent depression adjusting for age, gender, race/ethnicity, and household size, along with household income (model 2), household savings (model 3), homeownership (model 4), education (model 5), and marital status (model 6), respectively. Model 7 adjusted for all assets and demographic characteristics together. Third, we assessed the effect modification of assets on the relation between stressors and symptoms of persistent depression and tested for interaction. To do this, we estimated the predicted probability of symptoms of persistent depression by the stressors of job loss, financial difficulties, and relationship problems across each asset type, adjusting for demographic characteristics and for the interaction of each asset with each stressor. We used the margins command in STATA to calculate the predicted probabilities of the interaction term combinations. To estimate the predicted probabilities of symptoms of persistent depression on interaction pairs, we used unweighted regression models in our margin commands, as consistent with recommendations in the literature (*46*, *47*) and reported unweighted probabilities across interaction pairs for relevant comparison. We graphed the predicted probabilities in a figure with 15 panels representing each of the five asset and three stressor category combinations.

A correlation table across different asset types is listed in table S1, and a table with symptoms of persistent depression by asset types and corresponding *P* value is listed in table S2. A table with predicted probabilities and corresponding 95% CIs is listed in table 3. We conducted maximum likelihood ratio tests to measure the difference between each interaction term model and the relevant nested model with only main terms; we determined significance of the interaction terms using a *P* value cutoff of 0.05. Last, we calculated the prevalence of symptoms of persistent depression by interaction group categories to understand the direction of the associations. Maximum likelihood ratio test results and unadjusted prevalence of symptoms of persistent depression by asset and stressor groups are listed in table S4 and fig. S1, respectively. Use of survey weights is described in respective note of each figure and table. Analyses were conducted in STATA 16.1.
