# Supplementary material for: Assets, stressors, and symptoms of persistent depression over the first year of the COVID-19 pandemic
Source: Sci Adv. 2022 Mar 2;8(9):eabm9737. doi: 10.1126/sciadv.abm9737 (PMC8890702; doi:10.1126/sciadv.abm9737)
Supplement: Supplementary file 1 — Fig. S1 Tables S1 to S4 [file sciadv.abm9737_sm.pdf]

Science Advances
AAAS

Supplementary Materials for

Assets, stressors, and symptoms of persistent depression over the first year of the COVID-19 pandemic

Catherine K. Ettman*, Gregory H. Cohen, Salma M. Abdalla, Ludovic Trinquart, Brian C. Castrucci, Rachel H. Bork, Melissa A. Clark, Ira B. Wilson, Patrick M. Vivier, Sandro Galea

*Corresponding author. Email: cettman@bu.edu

Published 2 March 2022, *Sci. Adv.* **8**, eabm9737 (2022)
DOI: 10.1126/sciadv.abm9737

The PDF file includes:

Fig. S1
Tables S1 to S4

Other Supplementary Material for this manuscript includes the following:

Data S1

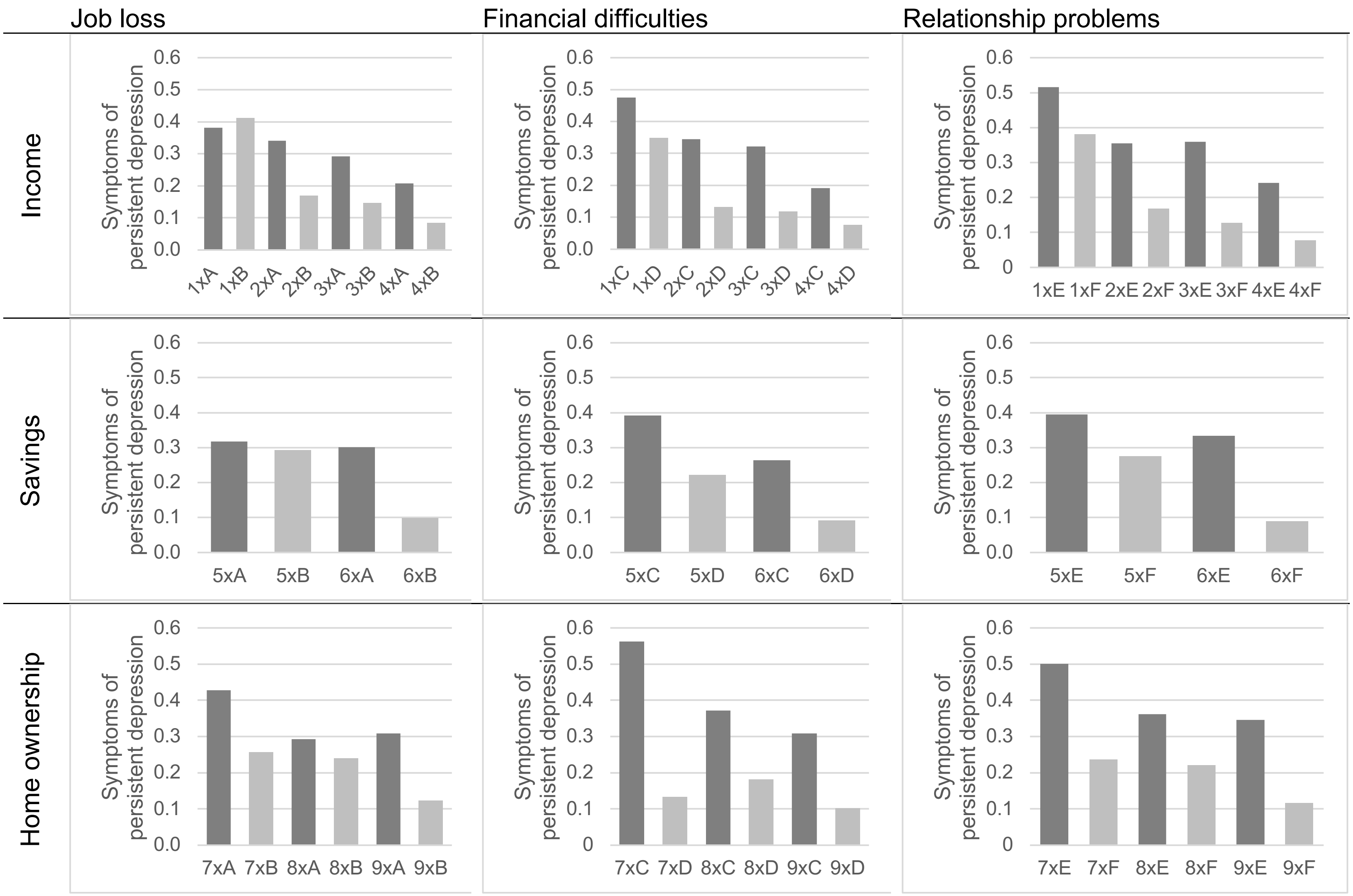

Job loss
Financial difficulties
Relationship problems
Income
Savings
Home ownership
Symptoms of persistent depression
0.0 0.1 0.2 0.3 0.4 0.5 0.6
1xA 1xB 2xA 2xB 3xA 3xB 4xA 4xB
1xC 1xD 2xC 2xD 3xC 3xD 4xC 4xD
1xE 1xF 2xE 2xF 3xE 3xF 4xE 4xF
5xA 5xB 6xA 6xB
5xC 5xD 6xC 6xD
5xE 5xF 6xE 6xF
7xA 7xB 8xA 8xB 9xA 9xB
7xC 7xD 8xC 8xD 9xC 9xD
7xE 7xF 8xE 8xF 9xE 9xF

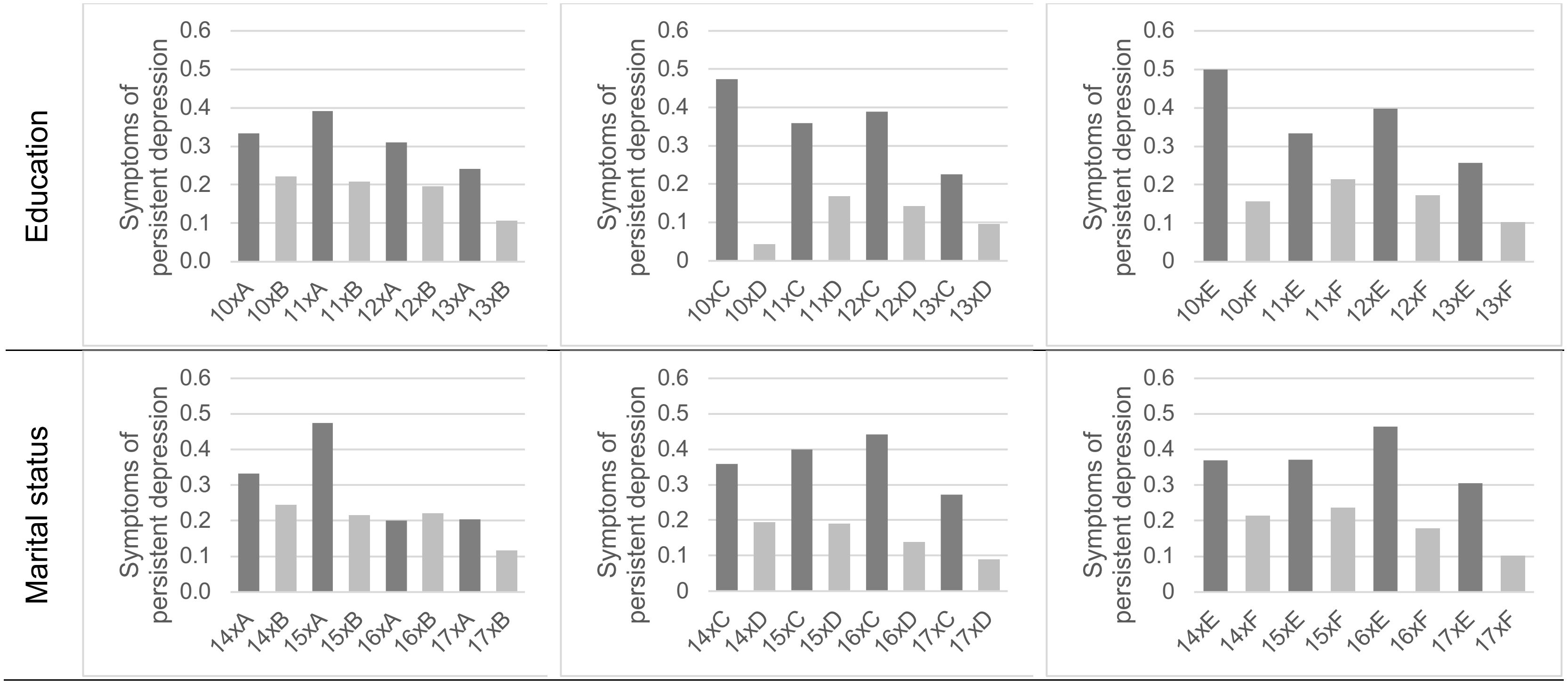

Fig. S1.

Supplemental Table 1. Correlation matrix for types of assets

Note: **Stressors**: A: Job loss, B: No job loss. C: financial difficulties; D: no financial difficulties. E: relationship problems; F: no relationship problems. **Assets: Household income**: 1: $0 - $19,999; 2: $20,000 - $44,999; 3: $45,000 - $74,999; 4: $75,000 or more. **Household savings**: 5: low savings (less than $5,000); 6: high savings ($5,000 or more). **Home ownership**: 7: Occupied without payment; 8: Home renter; 9; Home owner. **Education**: 10: No high school diploma; 11: High school graduate or equivalent; 12: Some college; 13: Bachelor's degree or above. **Marital status**: 14: Living with partner; 15: Never married; 16: Widowed, divorced, or separated; 17: Married. Time 1 stressors and assets reported. Persistent depression defined as presence of the Patient Health Questionnaire (PHQ)-9 score of 10 or greater at Time 1 and Time 2. Models adjusted for gender, age, race/ethnicity, and household size. Unweighted.

Table S1.

Prevalence of symptoms of persistent depression by asset and stressor interaction groups

	Household income	Household savings	Home ownership	Education	Marital status
Household income	1				
Household savings	0.49	1			
Home ownership	0.29	0.28	1		
Education	0.38	0.27	0.12	1	
Marital status	0.29	0.19	0.32	0.10	1

		Total		Symptoms of persistent depression		
		n	%	n	%	P-value
Financial assets	**Household income**					<0.001
	$0 - $19,999	167	19.0	68	40.9	
	$20,000 - $44,999	283	24.5	56	21.8	
	$45,000 - $74,999	297	27.2	47	16.1	
	≥$75,000	369	29.3	34	9.5	
	**Household savings**					<0.001
	$0 - $4,999	429	41.1	127	31.4	
	≥$5,000	680	59.0	78	13.2	
Physical assets	**Home ownership**					
	Occupied without payment	46	5.1	13	42.0	0.003
	Rented for cash	391	30.1	96	25.1	
	Home owner	702	64.8	99	16.3	
Social assets	**Education**					
	Less than high school graduate	42	9.3	10	25.5	0.015
	High school graduate or GED	206	28.1	47	26.4	
	Some college	506	27.7	106	22.8	
	College graduate or more	385	34.9	45	11.9	
	**Marital status**					
	Living with partner	106	9.6	27	28.8	
	Never married	258	27.4	66	27.6	
	Widowed, divorced, separated	196	16.2	43	22.9	
	Married	579	46.8	72	13.2	0.002

Table S2.

Symptoms of persistent depression in March-April 2020 (Time 1) and March-April 2021 (Time 2) by financial assets, physical assets, and social assets at Time 1.

Note: Time 1 assets reported. Column percentages provided for total; row percentages provided for persistent depression. Symptoms of persistent depression defined as presence of Patient Health Questionnaire (PHQ)-9 score of 10 or greater at both Time 1 and Time 2. N unweighted, % weighted using Time 2 survey weights.

	Job loss						Financial problems						Relationship problems					
	Yes			No			Yes			No			Yes			No		
	Pr	95%	CI	Pr	95%	CI	Pr	0.95	CI	Pr	95%	CI	Pr	95%	CI	Pr	95%	CI
**Household income**																		
$0 - $19,999	0.34	0.15	0.54	0.41	0.33	0.49	0.45	0.34	0.56	0.36	0.26	0.45	0.47	0.30	0.64	0.38	0.30	0.46
$20,000 - $44,999	0.33	0.20	0.47	0.18	0.13	0.23	0.34	0.24	0.44	0.14	0.09	0.19	0.34	0.20	0.47	0.18	0.13	0.23
$45,000 - $74,999	0.29	0.11	0.47	0.15	0.11	0.19	0.29	0.18	0.41	0.12	0.08	0.16	0.35	0.20	0.49	0.13	0.09	0.17
≥$75,000	0.18	0.04	0.32	0.08	0.05	0.11	0.18	0.08	0.28	0.07	0.05	0.10	0.21	0.08	0.35	0.08	0.05	0.10
**Household savings**																		
$0 - $4,999	0.29	0.18	0.40	0.28	0.23	0.33	0.37	0.30	0.44	0.21	0.16	0.27	0.36	0.25	0.46	0.27	0.22	0.31
≥$5,000	0.30	0.18	0.42	0.10	0.08	0.13	0.26	0.17	0.34	0.10	0.07	0.12	0.33	0.22	0.44	0.09	0.07	0.12
**Home ownership**																		
Occupied without payment	0.35	0.02	0.68	0.24	0.11	0.38	0.53	0.28	0.77	0.13	0.01	0.24	0.45	0.11	0.78	0.22	0.10	0.35
Rented for cash	0.27	0.14	0.40	0.23	0.19	0.28	0.35	0.27	0.43	0.18	0.13	0.22	0.33	0.23	0.44	0.21	0.17	0.26
Home owner	0.30	0.20	0.41	0.13	0.10	0.15	0.29	0.22	0.37	0.11	0.08	0.13	0.33	0.23	0.43	0.12	0.10	0.15
**Education**																		
Less than high school graduate	0.28	-0.05	0.61	0.23	0.09	0.36	0.47	0.25	0.70	0.04	-0.04	0.13	0.51	0.21	0.82	0.15	0.03	0.28
High school graduate or GED	0.36	0.17	0.54	0.22	0.16	0.28	0.36	0.24	0.48	0.18	0.12	0.25	0.35	0.16	0.54	0.22	0.16	0.29
Some college	0.28	0.17	0.39	0.20	0.16	0.24	0.36	0.28	0.44	0.15	0.11	0.19	0.36	0.26	0.45	0.18	0.14	0.21
College graduate or more	0.26	0.10	0.41	0.10	0.07	0.13	0.20	0.11	0.30	0.09	0.06	0.13	0.23	0.10	0.36	0.10	0.07	0.13
**Marital status**																		
Living with partner	0.28	0.04	0.51	0.23	0.15	0.31	0.32	0.18	0.46	0.18	0.09	0.28	0.34	0.17	0.51	0.20	0.11	0.28
Never married	0.44	0.29	0.59	0.21	0.16	0.27	0.37	0.27	0.48	0.19	0.13	0.25	0.33	0.18	0.48	0.23	0.18	0.29
Widowed, divorced, separated	0.22	0.00	0.44	0.23	0.17	0.30	0.43	0.29	0.56	0.15	0.09	0.21	0.47	0.28	0.65	0.19	0.13	0.25
Married	0.19	0.08	0.30	0.12	0.09	0.15	0.26	0.18	0.34	0.09	0.07	0.12	0.28	0.17	0.38	0.11	0.08	0.13

Table S3.

Predicted probability and 95% Confidence Interval (CI) of symptoms of persistent depression by job loss, financial problems, and relationship problems by assets.

Note: Predicted probability (Pr) shown. Adjusted for gender, age, race/ethnicity, and household size. GED: Graduate Equivalency Degree/General Educational Diploma. Symptoms of persistent depression defined as presence of the Patient Health Questionnaire (PHQ)-9 score of 10 or greater at both Time 1 and Time 2. Unweighted.

	Job loss	Financial problems	Relationship problems
	P-value	P-value	P-value
Household income	0.219	0.353	0.372
Household savings	**0.005**	0.264	**0.004**
Home ownership	0.151	0.375	0.318
Education	0.687	0.184	0.666
Marital status	0.347	0.561	0.397

Table S4.

Likelihood ratio test for the interaction between stressor and asset in Time 1 in their association with symptoms of persistent depression in Time 2.

Note: Data are p-values for the interaction between each stressor in column and each asset in rows. Interaction was tested by likelihood ratio tests comparing models with and without the corresponding interaction term. All models were further adjusted for gender, age, race/ethnicity, and household size. Unweighted. Bold indicates significance at 0.05. Unweighted.
